# Omissions from a National Institute of Health (NIH) biosketch

**DOI:** 10.1371/journal.ppat.1006896

**Published:** 2018-05-10

**Authors:** Alice Prince

**Affiliations:** College of Physician & Surgeons, Columbia University, New York, New York, United States of America; The Fox Chase Cancer Center, UNITED STATES

The standard National Institute of Health (NIH) biosketch provides a snapshot of an academic’s achievements, but mine, like most, lists only the highpoints and not many of the other major factors that influenced my career in academic medicine. A review of some notable omissions may provide some useful insights. A molecular biology major in college, my several summers of bench research on cyanobacteria probably helped get me into medical school by offsetting the Cs in physics and organic chemistry (Wellesley didn’t do grade inflation). I did not appreciate that all those pathways require rote memorization, a skill I picked up later, which is now made unnecessary by Wikipedia. Nor does my biosketch include the hours I spent poring over the writings of Peter Kropotkin and Emma Goldman while struggling with my own personal involvement in the turbulent political scene of the ‘70s. That was simply listed as “honors research in political philosophy.” Once in medical school, also amidst a background of political upheaval, I sought out a summer research position in a lab, which in retrospect provided a setting in which I had some control. Bench research provided a sense of rationality and order: testable hypotheses, controls, and tangible outcomes, some of which were actually publishable. The social and political issues then, as now, seemed insurmountable. This part of my biosketch that delineates postgraduate training omits all of the distractions of ongoing political turmoil and suggests I learned molecular biology skills as well as clinical infectious diseases. It fails to mention the most notable achievement of my fellowships, namely the births of my sons.

The next portion of my biosketch is remarkably unidimensional as I have remained at one institution for virtually all of my academic career. Although this is considered “undesirable”, it was expedient. When trying to juggle two professional careers, stable day care, a strong public school system, room for a dog, and the requirement to live east of the Hudson River and north of 165th street, the options become limited. The support of family and friends is a high priority for two-career couples, particularly when there are so many arbitrary external constraints. I was able to find ample diversity and inspiration among willing collaborators in cell biology, immunology, and pharmacology. My biosketch does reflect the multidisciplinary nature of my research, which was launched by a local colleague who offered to share his bovine tracheal epithelial cells freshly obtained from Max the kosher butcher for our studies to identify *Pseudomonas aeruginosa* receptors in the airway.

Research funding is also listed on a standard biosketch, and again I look fine, now. It does not list the innumerable failed attempts or the close misses at grant funding. The critique suggesting that my central hypothesis (long since published in highly esteemed journals) was “fundamentally flawed” is not mentioned, nor are the comments noting that I was a “lady pediatrician” who should perhaps return to weighing babies. My colleagues, at least some of them, will confess to similarly devastating reviews. However, we are all driven by our fundamental belief that our work is important and by varying degrees of arrogance that spur us to try again, and again, and again.

Perhaps the greatest omission from my biosketch is a sense of the enthusiasm I have in exploring a range of topics related to host—pathogen interactions. It does not point out the students who graciously put up with emails on Christmas day when I had an idea that couldn’t wait or those who agreed to try an experimental approach that seemed doomed to failure. In return, I also appreciate the many topics that I would never have broached without their insistence (connexins, Ca^2+^ signaling, and even keratinocyte biology). Also not explicitly listed are the contributions from friends met at meetings or at grant reviews or online who provided mutant mouse femurs for stem cell isolation or constructed and complemented mutants that we failed to produce ourselves, some of which were important additions to the experimental data and others only to satisfy a cranky reviewer. Although our studies to determine why *P*. *aeruginosa* infects cystic fibrosis (CF) patients began by using classical bacterial and murine genetics—well within my own skill set—more recent approaches have required major collaborations in genomics, metabolomics, and the bioinformatic approaches necessary to use this data intelligently. While I do have insights into which components of our data seem most relevant to clinical medicine as it’s currently being practiced, I rely upon the talents of numerous individuals to help generate a coherent data set from a sketchy hypothesis. I am always amazed at how such disparate individuals brought together by a shared intellectual curiosity can accomplish so much. My biosketch hopefully does reflect what can be accomplished by convening a group of creative and unselfish individuals with common goals, occasional parties, and a reliable source of bagels.

**Image 1:**
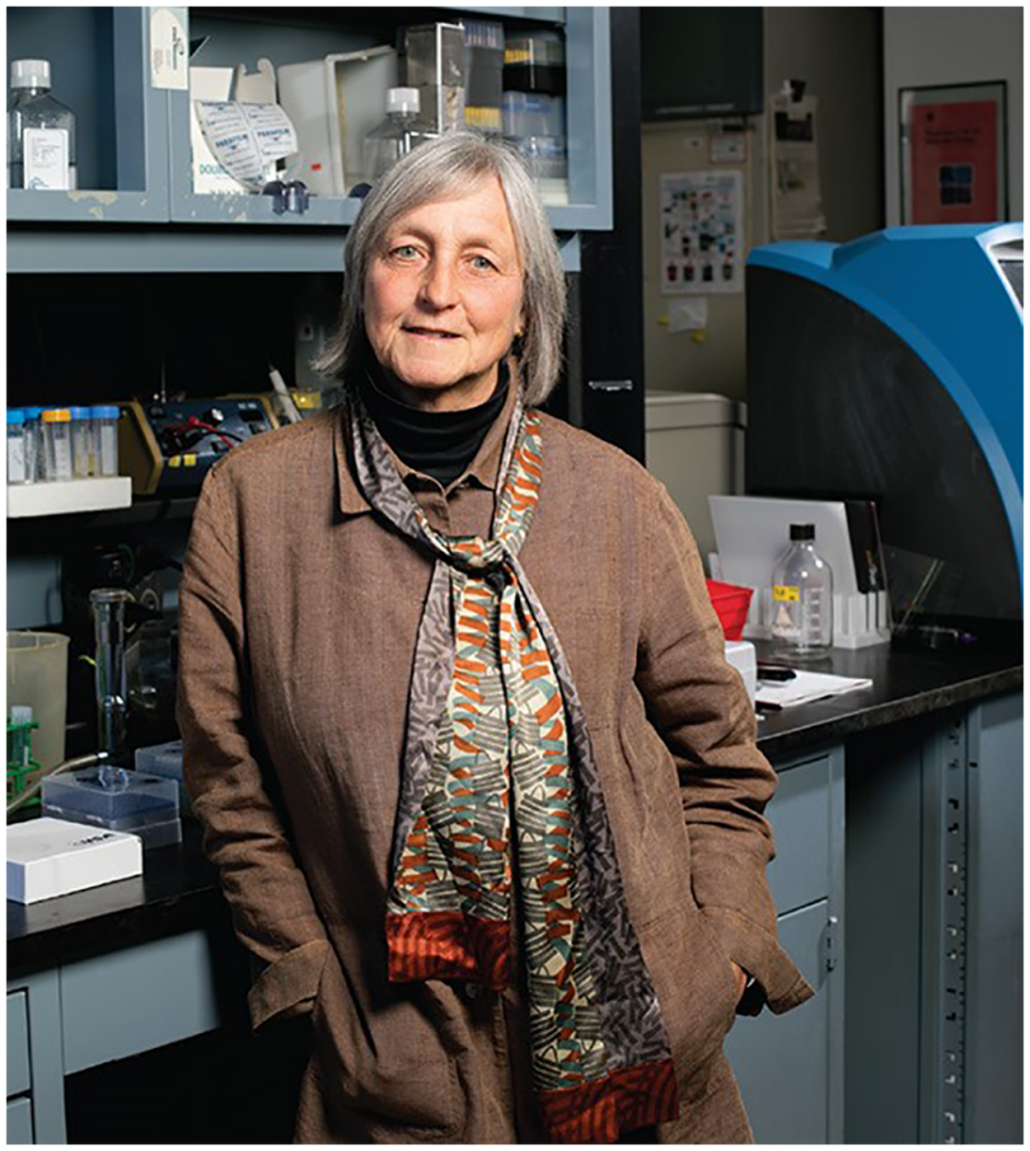
Alice Prince.

